# Accuracy of Intraocular Lens Power Calculation Formulas in Pediatric Cataract Patients: A Systematic Review and Meta-Analysis

**DOI:** 10.3389/fmed.2021.710492

**Published:** 2021-11-26

**Authors:** Yueyang Zhong, Yibo Yu, Jinyu Li, Bing Lu, Su Li, Yanan Zhu

**Affiliations:** School of Medicine, Eye Center of the Second Affiliated Hospital, Zhejiang University, Hangzhou, China

**Keywords:** pediatric cataract, calculation formula, intraocular lens power, prediction error, meta-analysis

## Abstract

**Background:** Among the various intraocular lens (IOL) power calculation formulas available in clinical settings, which one can yield more accurate results is still inconclusive. We performed a meta-analysis to compare the accuracy of the IOL power calculation formulas used for pediatric cataract patients.

**Methods:** Observational cohort studies published through April 2021 were systematically searched in PubMed, Web of Science, and EMBASE databases. For each included study, the mean differences of the mean prediction error and mean absolute prediction error (APE) were analyzed and compared using the random-effects model.

**Results:** Twelve studies involving 1,647 eyes were enrolled in the meta-analysis, and five formulas were compared: Holladay 1, Holladay 2, Hoffer Q, SRK/T, and SRK II. Holladay 1 exhibited the smallest APE (0.97; 95% confidence interval [CI]: 0.92–1.03). For the patients with an axial length (AL) less than 22 mm, SRK/T showed a significantly smaller APE than SRK II (mean difference [MD]: −0.37; 95% CI: −0.63 to −0.12). For the patients younger than 24 months, SRK/T had a significantly smaller APE than Hoffer Q (MD: −0.28; 95% CI: −0.51 to −0.06). For the patients aged 24–60 months, SRK/T presented a significantly smaller APE than Holladay 2 (MD: −0.60; 95% CI: −0.93 to −0.26).

**Conclusion:** Due to the rapid growth and high variability of pediatric eyes, the formulas for IOL calculation should be considered according to clinical parameters such as age and AL. The evidence obtained supported the accuracy and reliability of SRK/T under certain conditions.

**Systematic Review Registration:** PROSPERO, identifier: INPLASY202190077.

## Introduction

Pediatric cataract accounts for 5–20% of the global cases of childhood blindness (1). The etiology of pediatric cataract is diverse, which includes genes mutations and various disruptive factors during the embryonic to postnatal stages (2). With the development of modern diagnostic technology, cataract surgical techniques and intraocular lens (IOL) designs, pediatric cataract surgery has been recommended as a safe and effective intervention for optical correction in infants and young children (1, 3, 4). Nevertheless, controversies still exist on the timing of intervention, IOL implantation, and postoperative management for pediatric cataract. Even with a successful cataract surgery, it is still challenging to achieve the desired refractive outcomes in children. Pediatric eyes generally have shorter axial lengths (ALs), higher keratometry values, and smaller anterior chamber depths (ACD). Distinct from the eyes of an adult, pediatric eyes are characterized by rapid growth and constantly varying parameters, which may result in significant refractive change during the postoperative optical rehabilitation (3, 5). Aside from that, the inaccurate measurements of the parameters of children due to poor cooperation and fixation also complicates the calculation of IOL power for pediatric cataract patients.

At present, the IOL power calculation formulas applied in pediatric patients were derived from the data of adult eyes, which may be impractical for application in the eyes of children. The second-generation SRK II formula is a regression formula adjusted for AL and keratometry that was commonly used (6). Subsequently, the third-generation formula of Holladay 1 (7) and Hoffer Q (8) formulas introduced ACD and corneal curvature into the calculation. Another third-generation formula, SRK/T (9), is a non-linear theoretical formula optimized for postoperative ACD, retinal thickness, AL, and corneal refractive index. Thereafter, Holladay 2, a fourth-generation formula, takes the effective lens position and characteristics of patients into account to achieve personalized calculation (10). In adults, the Holladay 2 formula has been considered as the most accurate for eyes with an AL of 22–26 mm (11). The Haigis formula showed superiority over other formulas in short eyes of the adults (AL <22 mm) (12), while the SRK/T is considered optimal for long eyes (AL > 26 mm) (13).

Although the predictability of the IOL formulas for adults has been studied extensively, controversies still exist regarding the most appropriate IOL calculation formula for pediatric cataract patients (12, 14–17). For example, Andreo et al. (18) and Neely et al. (19) did not report a significant association between IOL formulas and refractive prediction error. On the other hand, Nihalani and Vanderveen (20) suggested that Hoffer Q was the most accurate formula, whereas Kekunaya et al. (21) found that the SRK II formula was superior to the other formulas. To date, the comparisons of the predictability of the IOL calculation formulas for pediatric cataract patients have yielded inconsistent results. We therefore conducted an initial and comprehensive systematic review and meta-analysis to evaluate and compare the refractive prediction performances of the different IOL power calculation formulas in pediatric cataract patients.

## Materials and Methods

This meta-analysis was designed, implemented, and performed in accordance with the meta-analysis of observational studies in epidemiology (MOOSE) protocol (22), and is reported herein following the preferred reporting items for systematic reviews (PRISMA) guidelines (23). The protocol for this systematic review was registered on International Platform of Registered Systematic Review and Meta-analysis Protocols (INPLASY) (registration number: INPLASY202190077).

### Search Strategy

Two independent investigators (YZ and YY) systematically searched the databases of PubMed, Web of Science, and EMBASE for cohort studies published through April, 2021. The following search strategy was used: (pediatric cataract) AND (calculate OR formula) AND (IOL OR IOL). Only articles published in English and full-text journal articles of original studies were included. Furthermore, the references cited in the relevant articles were reviewed for additional eligible publications.

### Eligibility Criteria

Studies that met the following criteria were included in our meta-analysis: (i) included pediatric cataract patients who underwent cataract extraction and primary posterior chamber IOL implantation; (ii) compared at least two types of the target IOL power calculation formula (Holladay 1, Holladay 2, Hoffer Q, SRK/T, and SRK II); and (iii) provided either prediction error (PE) or APE values (with 95% confidence intervals [CIs]). We excluded reviews, non-comparative studies, case reports, studies that lacked sufficient data, and other non-relevant publications.

### Data Extraction

Two independent investigators (YZ and YY) conducted an initial screening of titles and abstracts and then evaluated the full texts of the eligible studies. Any discrepancies were resolved through group discussion. The data were extracted in a standardized data collection form including the following information from each included study: first author, year of publication, study location, study design, sample size, gender, age, AL, follow-up duration, IOL calculation formulas used, and the PE and/or APE values with their 95% CIs.

### Quality Assessment

Quality assessment was performed using the revised quality assessment of diagnostic accuracy studies (QUADAS-2) tool (24). QUADAS-2 is applied in four phases: summarizing the review question, tailoring the tool and producing review-specific guidance, constructing a flow diagram for the primary study, and judging bias and applicability. For judgments of risk of bias and applicability, four domains discussing patient selection, index test, reference standard, and flow and timing were assessed with 14 signaling questions.

### Statistical Analysis

In this meta-analysis, PE and/or APE with 95% CI was considered as the common measure of comparison of the different IOL calculation formulas across studies. The random-effects model (DerSimonian-Laird method) was used to calculate the summarized mean differences (MDs) and their corresponding 95% CIs (25, 26).

The heterogeneity among the studies was estimated using the *I*^2^ statistic, with cutoff values representing low (25%), moderate (50%), and high (75%) degrees of heterogeneity (27). To explore the potential confounding factors, we performed subgroup analyses based on different ages and ALs.

### Sensitivity Analysis and Publication Bias

Sensitivity analyses were performed by omitting one study at a time and calculating a pooled estimate for the remainder of the studies to determine if the results were markedly affected by a single study. The publication bias was evaluated by the application of Egger's linear regression test and Begg's rank correlation test with the significance set at *p* < 0.10 (28, 29). All the statistical analyses were performed using Stata (version 15.0; StataCorp LP, College Station, TX, USA). All the tests were two tailed, and differences with *p* < 0.05 were considered significant.

## Results

### Search Process

**Figure 1:**
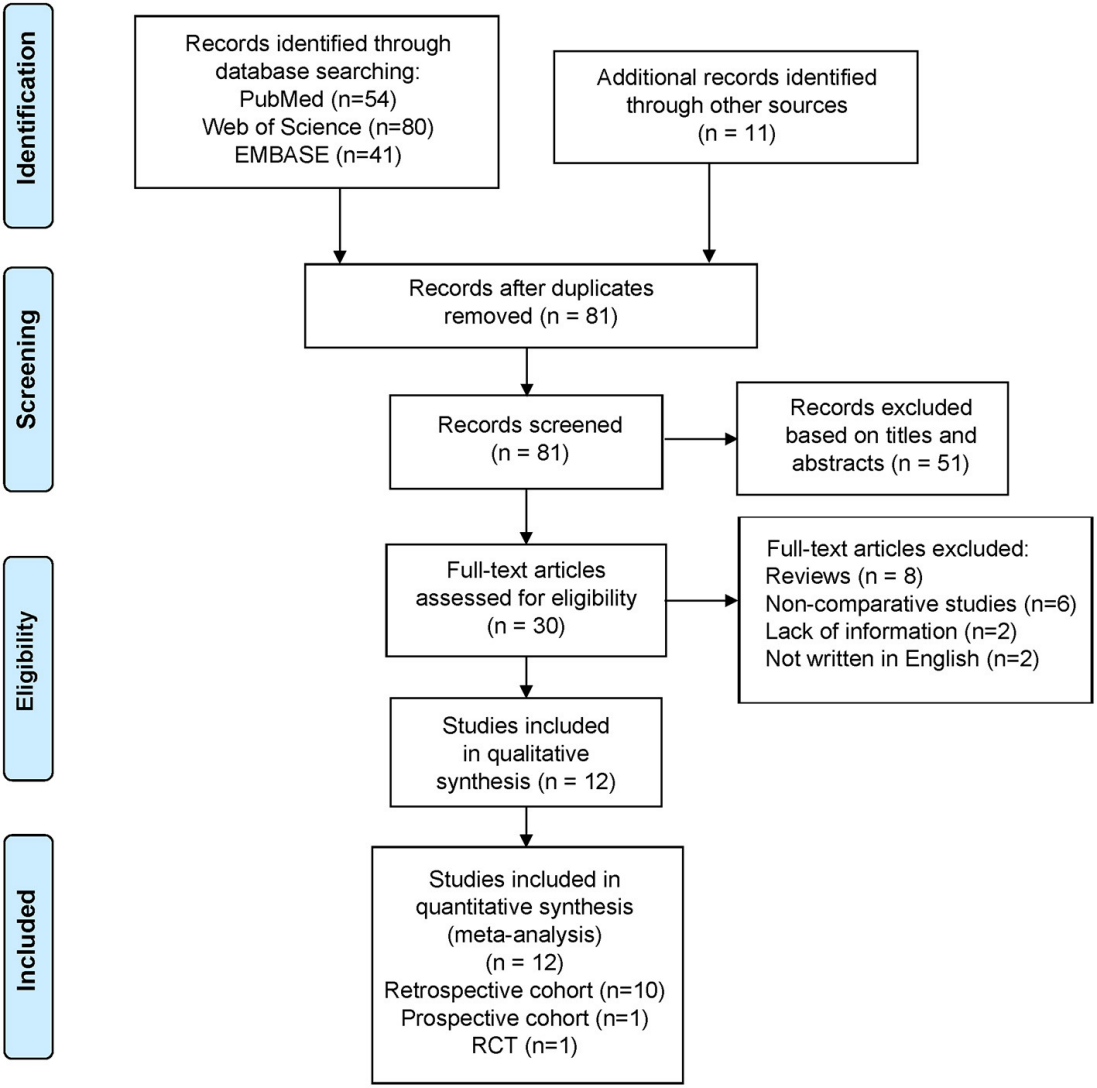
Flowchart depicting the literature search and selection strategy.

Of the 186 articles identified (54 from PubMed, 80 from Web of Science, 41 from EMBASE, and 11 from other sources), we excluded 105 duplicates and 69 studies that did not meet the aforementioned criteria (Figure 1). Eventually, we included 12 studies in the meta-analysis (11 cohort studies and one randomized comparative trial).

### Study Characteristics

**Figure 2:**
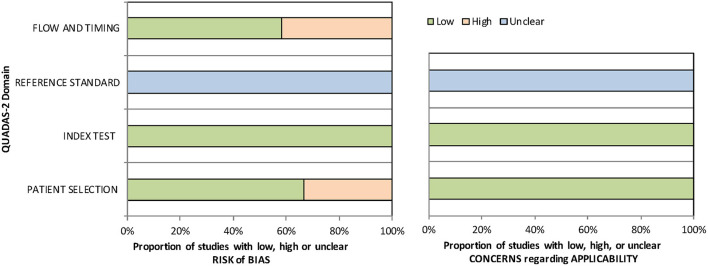
QUADAS-2 quality assessment of the included studies.

Table 1 summarizes the descriptive characteristics of the included studies. A total of 1,647 eyes from 12 studies were enrolled in our meta-analysis (20, 21, 30–39). Five of the studies were conducted in the United States, three in China, three in other Asian countries, and one in Canada. Ten studies (1,102 eyes) assessed the predictability of IOL calculation formula with Holladay 1, six studies (439 eyes) with Holladay 2, 12 studies (1,647 eyes) with Hoffer Q and SRK/T, and eight studies (1,319 eyes) with SRK II. The quality assessment results using the QUADAS-2 tool indicated a low risk of bias of the included studies (Supplementary Table 1 and Figure 2).

**Table 1:** Characteristics of the included studies (*n* = 12).

**References**	**Country**	**Study design**	**Eyes** **(n)**	**Sex** **(M/F)**	**Age (months)^**a**^**	**Axial length (mm)^**b**^**	**Measurement (weeks)^**c**^**	**IOL power calculation formulas**
Eppley et al., (30)	US	Retrospective cohort	64	32/32	70.8 ± 42.7 (17.5–185.4)	22.6 ± 1.6 (19.6–26.3)	7.2 ± 3.6 (3-16)	Holladay 2, Hoffer Q, SRK/T, Barrett
Chang et al., (31)	China	Retrospective cohort	68	35/33	34.1 ± 24.6	21.1 ± 1.4	4	Holladay 1, Holladay 2, Hoffer Q, SRK/T, SRK II, Haigis, Barrett
Kou et al., (39)	China	Prospective cohort	102	NA	41.4 (6-84)	21.8 (18.1–25.9)	4	Holladay 1, Holladay 2, Hoffer Q, SRK/T, Haigis
Li et al., (32)	China	Retrospective cohort	377	194/183	55.2 ± 28.0 (9-150)	21.5 ± 1.9 (17.9–31.5)	6.0 ± 2.7 (3.7–14.4)	Holladay 1, Hoffer Q, SRK/T, SRK II
Lee et al., (33)	Korea	Retrospective cohort	481	182/156	43.6 ± 30.1 (11-210)	21.3 ± 1.7 (15.2–27.5)	4–10	Hoffer Q, SRK/T, SRK II
Vasavada et al., (34)	US	Retrospective cohort	117	NA	35.6 ± 35.6 (2.4–165.6)	20.9 ± 2.8 (17.1–26.1)	4.0 ± 2.4 (2.0–6.8)	Holladay 1, Holladay 2, Hoffer Q, SRK/T
Joshi et al., (35)	Nepal	Retrospective cohort	28	13/6	79.2 ± 48.0 (24-168)	19.2 ± 0.9 (17.1–20.0)	> 6	Holladay 1, Hoffer Q, SRK/T, SRK II
Vanderveen et al., (36)	US	RCT	43	NA	2.5 ± 1.5	18.1 ± 1.1	4	Holladay 1, Holladay 2, Hoffer Q, SRK/T, SRK II
Kekunnaya et al., (21)	India	Retrospective cohort	128	41/43	11.7 ± 6.2 (1.5–23)	19.9 ± 1.7 (16.3–25.7)	4	Holladay 1, Hoffer Q, SRK/T, SRK II
Trivedi et al., (37)	US	Retrospective cohort	45	NA	46.8 ± 34.8 (1.2–124.8)	21.7 ± 2.0 (16.8–27.6)	7.8 ± 2.8 (3.8–15.0)	Holladay 1, Holladay 2, Hoffer Q, SRK/T
Nihalani and VanderVeen, (20)	US	Retrospective cohort	135	51/45	76.8 (1.1–216)	22.2 (17.7–27.8)	4–8	Holladay 1, Hoffer Q, SRK/T, SRK II
Mezer et al., (38)	Canada	Retrospective cohort	59	34/15	89.0 (22-216)	26.7 (19.2–26.7)	8–24	Holladay 1, Hoffer Q, SRK/T, SRK II

*M, Male; F, Female; IOL, intraocular lens; RCT, randomized comparative trial; NA, not available*.

a *Age: mean ± standard deviation (SD) and/or range*.

b *Axial length: mean ± SD and/or range*.

c *Measurement: mean ± SD and/or range*.

### Comparison of the APEs of the Different Formulas

The APEs of the studied formulas are shown in Supplementary Figure 1. All the formulas showed inevitable APE in pediatric eyes. Holladay 1 demonstrated the smallest APE (0.97; 95% CI: 0.92–1.03), followed by Holladay 2 (APE: 1.05; 95% CI: 0.94–1.17) and Hoffer Q (APE: 1.05; 95% CI: 1.00–1.11). Among all the studied formulas, SRK II showed the largest APE of (1.34; 95% CI: 1.28–1.41). As shown in Table 2, the MDs of PEs, APEs, and relative risks of APE < 0.5D of the different formulas were not significant. However, the pooled results were characterized by substantial heterogeneity and warrants further subgroup analysis.

**Table 2:** Mean difference of prediction error (PE), absolute prediction error (APE), and relative risk of APE prediction < 0.5 D.

**Formula comparison**	**PE**			**APE**			**APE prediction** **<** **0.5 D**		
	**N**	**MD (95% CI)**	***I^**2**^***	**N**	**MD (95% CI)**	***I^**2**^***	**N**	**RR (95% CI)**	***I^**2**^***
Holladay 1 vs. Holladay 2	3	0.03 (−0.22, 0.29)	45.1%	4	−0.06 (−0.23, 0.11)	0	2	0.98 (0.86, 1.11)	0
Holladay 1 vs. Hoffer Q	7	−0.05 (−0.22, 0.12)	63.2%	9	−0.11 (−0.23, 0.01)	35.2%	5	0.99 (0.94, 1.06)	0
Holladay 1 vs. SRK/T	7	0.01 (−0.11, 0.13)	30.7%	9	−0.01 (−0.09, 0.08)	0	5	1.00 (0.94, 1.07)	0
Holladay 1 vs. SRK II	5	0.02 (−0.41, 0.45)	92.8%	7	−0.07 (−0.40, 0.27)	89.4%	4	1.03 (0.96, 1.10)	60.9%
Holladay 2 vs. Hoffer Q	4	−0.18 (−0.47, 0.01)	26.2%	4	−0.08 (−0.33, 0.18)	53.8%	2	1.04 (0.91, 1.19)	0
Holladay 2 vs. SRK/T	4	0.10 (−0.16, 0.35)	58.3%	4	0.13 (−0.13, 0.39)	55.0%	3	1.03 (0.93, 1.14)	0
Hoffer Q vs. SRK/T	9	0.17 (−0.07, 0.40)	88.3%	10	0.17 (−0.01, 0.35)	79.8%	5	1.01 (0.95, 1.07)	0
Hoffer Q vs. SRK II	6	0.17 (−0.35, 0.69)	97.1%	8	0.12 (−0.24, 0.48)	94.4%	4	1.04 (0.97, 1.11)	74.0%
SRK/T vs. SRK II	6	0.04 (−0.21, 0.28)	86.8%	8	−0.09 (−0.30, 0.13)	84.0%	4	1.02 (0.95, 1.09)	67.8%

*MD, mean difference; 95% CI, 95% confidence interval; RR, relative risk*.

**Figure 3:**
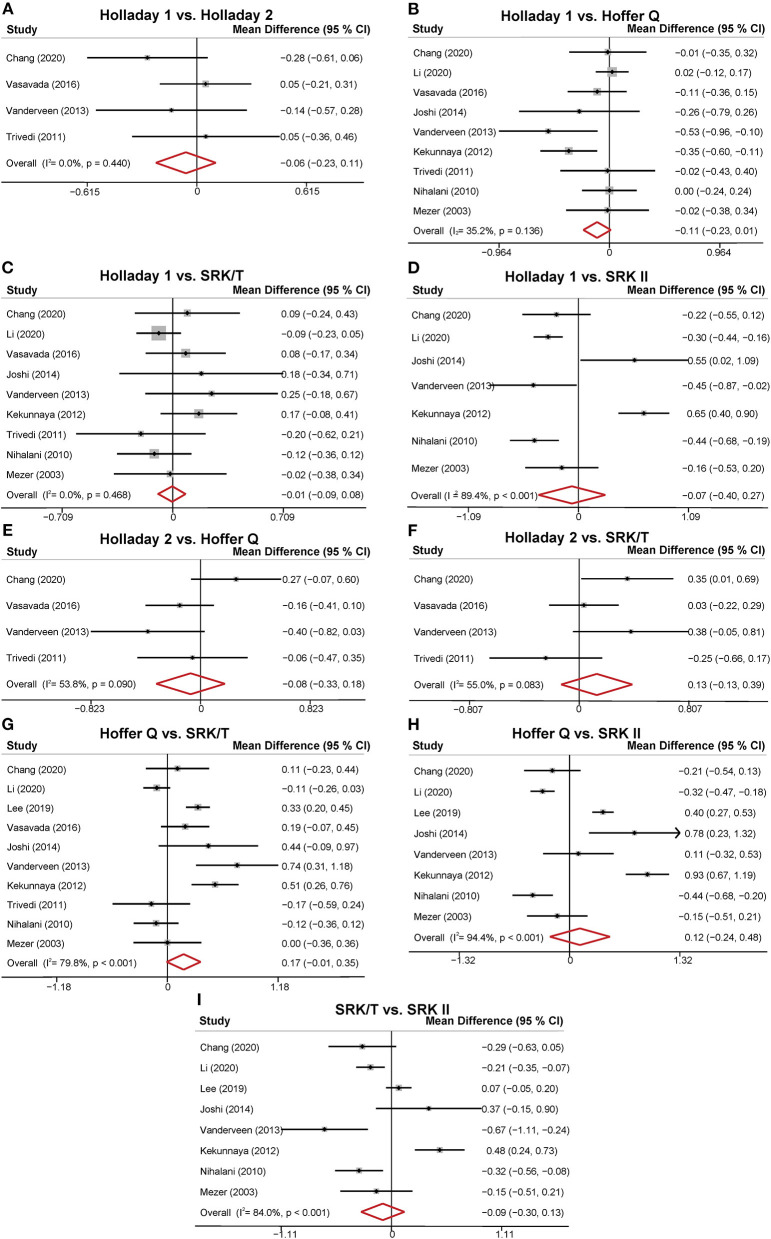
Forest plot of the mean difference (MD) of absolute prediction error (APE) of the different formulas. **(A)** Holladay 1 vs. Holladay 2; **(B)** Holladay 1 vs. Hoffer Q; **(C)** Holladay 1 vs. SRK/T; **(D)** Holladay 1 vs. SRK II; **(E)** Holladay 2 vs. Hoffer Q; **(F)** Holladay 2 vs. SRK/T; **(G)** Hoffer Q vs. SRK/T; **(H)** Hoffer Q vs. SRK II; **(I)** SRK/T vs. SRK II.

The MDs of the APEs of the different formulas are presented in Figure 3. Four studies compared the predictability levels of Holladay 1 and Holladay 2. The APEs of these formulas were not significantly different (MD: −0.06; 95% CI: −0.23 to 0.11; Figure 3A), with low heterogeneity (*I*^2^ = 0). Nine studies compared the APEs of Holladay 1 and Hoffer Q. The pooled results indicated no significant difference in APEs (MD: −0.11; 95% CI: −0.23 to 0.01; Figure 3B), with moderate heterogeneity (*I*^2^ = 35.2%). Sensitivity analysis was conducted by omitting one study at a time, and after excluding the study by Kekunnaya et al. (21) the heterogeneity decreased significantly (from 35.2%−0). However, the APEs of Holladay 1 and Hoffer Q were still comparable (MD: −0.04; 95% CI: −0.13 to 0.05; Supplementary Table 2). In addition, after excluding the study by Li et al. (32), the APE became significantly smaller with Holladay 1 than with Hoffer Q (MD: −0.15; 95% CI: −0.27 to −0.02; Supplementary Figure 2). The nine studies comparing the Holladay 1 and SRK/T formulas showed no significant difference in APE (MD: −0.01; 95% CI: −0.09 to 0.08; Figure 3C), and no heterogeneity (*I*^2^ = 0) was detected for the pooled analysis. For Holladay 1 and SRK II, seven studies were analyzed, and the APEs of the groups were found to be similar (MD: −0.07; 95% CI: −0.40 to 0.27; Figure 3D) with high heterogeneity (*I*^2^ = 89.4%). Nevertheless, when the study by Kekunnaya et al. (21) was excluded, the heterogeneity decreased from 89.4 to 58.1% and the pooled results indicated a significantly smaller APE with Holladay 1 than with SRK II (MD: −0.23; 95% CI: −0.43 to −0.04; Supplementary Figure 2).

Four studies compared the predictability levels of Holladay 2 and Hoffer Q. No significant difference was found in the groups' APEs (MD: −0.08; 95% CI: −0.33 to 0.18; Figure 3E) with moderate heterogeneity (*I*^2^ = 53.8%). The sensitivity analysis suggested that the study by Chang et al. (31) was the source of heterogeneity. The heterogeneity decreased to 0 when the study by Chang et al. (31) was excluded (MD: −0.18; 95% CI: −0.38 to 0.01; Supplementary Table 2). The four studies comparing the Holladay 2 and SRK/T formulas showed no significant difference in APE (MD: 0.13; 95% CI: −0.13 to 0.39; Figure 3F), and moderate heterogeneity (*I*^2^ = 55.0%) was detected. Ten studies were included to compare the APEs with Hoffer Q and with SRK/T, and no significant difference was found (MD: 0.17; 95% CI: −0.01 to 0.35; Figure 3G). After removing the study by Li et al. (32), the between-study heterogeneity decreased from 79.8% to 69.9%, and the SRK/T formula presented a significantly smaller APE than the Hoffer Q formula (MD: −0.21; 95% CI: −0.39 to −0.04; Supplementary Figure 2). With respect to Hoffer Q and SRK II formulas, eight studies were included in the meta-analysis. No significantly different APE was found (MD: 0.12; 95% CI: −0.24 to 0.48; Figure 3H) with substantial heterogeneity (*I*^2^ = 94.4%). The APEs with SRK/T and with SRK II formulas were compared in eight studies and were not significantly different (MD: −0.09; 95% CI: −0.30 to 0.13; Figure 3I). Substantial between-study heterogeneity was detected (*I*^2^ = 84.0%).

### Subgroup Analyses

Subgroup analyses were performed based on different ranges of age and AL. As shown in Table 3, three studies compared the PEs of Holladay 2 and Hoffer Q in patients with an AL smaller than 22 mm. The pooled results indicated a significantly smaller PE with Holladay 2 than with Hoffer Q (MD: −0.37; 95% CI: −0.65 to −0.10), with no between-study heterogeneity. In addition, for the patients with an AL of 22–24.5 mm, a significantly larger PE was found with Hoffer Q than with SRK/T (MD: 0.25; 95% CI: 0.06 to 0.44).

**Table 3:** Mean difference of prediction error (PE) between groups stratified by age and axial length.

**Formula comparison**	**AL** **<** **22 mm**			**AL 22**–**24.5 mm**			**Age** **<** **24 months**			**Age 24**–**60 months**		
	**N**	**MD (95% CI)**	***I^**2**^***	**N**	**MD (95% CI)**	***I^**2**^***	**N**	**MD (95% CI)**	***I^**2**^***	**N**	**MD (95% CI)**	***I^**2**^***
Holladay 1 vs. Holladay 2	3	0.05 (−0.24, 0.35)	13.0%	2	−0.10 (−0.52, 0.32)	0	2	0.06 (−0.27, 0.40)	0	2	0.23 (−0.25, 0.71)	51.7%
Holladay 1 vs. Hoffer Q	4	−0.14 (−0.50, 0.22)	68.8%	3	−0.13 (−0.32, 0.06)	0	4	0.15 (−0.10, 0.39)	34.8%	3	0.09 (−0.08, 0.26)	0
Holladay 1 vs. SRK/T	4	0.03 (−0.24, 0.29)	44.7%	3	0.12 (−0.07, 0.31)	0	4	−0.11 (−0.29, 0.08)	0	3	−0.09 (−0.25, 0.08)	0
Holladay 1 vs. SRK II	2	−0.09 (−0.86, 0.67)	89.2%	NA			3	−0.27 (−0.91, 0.37)	87.6%	2	−0.12 (−0.51, 0.26)	58.0%
Holladay 2 vs. Hoffer Q	3	**−0.37 (−0.65**, −0.10)^*^	0	2	0.03 (−0.39, 0.45)	0	2	−0.07 (−0.72, 0.57)	73.1%	2	−0.24 (−1.06, 0.58)	83.1%
Holladay 2 vs. SRK/T	3	0.15 (−0.23, 0.52)	44.9%	2	0.27 (−0.15, 0.70)	0	2	0.02 (−0.32, 0.35)	0	2	−0.20 (−0.93, 0.53)	78.9%
Hoffer Q vs. SRK/T	4	0.26 (−0.36, 0.88)	89.4%	3	0.25 (0.06, 0.44)^*^	0	4	−0.16 (−0.55, 0.23)	73.9%	2	0.04 (−0.29, 0.37)	0
Hoffer Q vs. SRK II	2	−0.07 (−1.30, 1.16)	95.7%	NA			3	−0.30 (−1.20, 0.61)	93.7%	NA		
SRK/T vs. SRK II	2	−0.07 (−0.49, 0.34)	65.4%	NA			NA			NA		

*AL, axial length; MD, mean difference; 95% CI, 95% confidence interval; NA, not available*.

* *Indicates statistical significance*.

The results of the subgroup analyses of APEs based on different ranges of age and AL are presented in Table 4. For the patients younger than 24 months, a significantly smaller APE was found with SRK/T than with Hoffer Q (MD: −0.28; 95% CI: −0.51 to −0.06). For the patients aged between 24 and 60 months, Holladay 1 formula showed a smaller APE than SRK II (MD: −0.30; 95% CI: −0.48 to −0.12). Furthermore, Holladay 2 formula showed a significantly higher APE than Hoffer Q (MD: 0.54; 95% CI: 0.21 to 0.88) and SRK/T (MD: 0.60; 95% CI: 0.26 to 0.93). When stratified by AL, a significantly smaller APE was found with Holladay 1 than with SRK II (MD: −0.39; 95% CI: −0.56 to −0.21) among the patients with an AL smaller than 22 mm. SRK/T exhibited better predictability than SRK II (MD: −0.37; 95% CI: −0.63 to −0.12).

**Table 4:** Mean difference of absolute prediction error (APE) between groups stratified by age and axial length.

**Formula comparison**	**AL** **<** **22 mm**			**AL 22**–**24.5 mm**			**Age** **<** **24 months**			**Age 24**–**60 months**		
	**N**	**MD (95% CI)**	***I^**2**^***	**N**	**MD (95% CI)**	***I^**2**^***	**N**	**MD (95% CI)**	***I^**2**^***	**N**	**MD (95% CI)**	***I^**2**^***
Holladay 1 vs. Holladay 2	4	−0.09 (−0.33, 0.14)	0	2	0.15 (−0.28, 0.57)	0	2	−0.25 (−0.58, 0.09)	0	2	−0.25 (−0.58, 0.08)	0
Holladay 1 vs. Hoffer Q	5	−0.15 (−0.38, 0.08)	41.6%	3	0.02 (−0.17, 0.21)	0	4	−0.18 (−0.38, 0.03)	14.4%	3	0.15 (−0.15, 0.46)	51.6%
Holladay 1 vs. SRK/T	5	−0.01 (−0.19, 0.16)	11.2%	3	−0.04 (−0.29, 0.21)	15.9%	4	0.11 (−0.08, 0.29)	0	3	0.15 (−0.26, 0.56)	72.4%
Holladay 1 vs. SRK II	3	**−0.39 (−0.56**, −0.21)^*^	0	NA			3	0.08 (−0.58, 0.74)	88.6%	2	**−0.30 (−0.48**, −0.12)^*^	0
Holladay 2 vs. Hoffer Q	4	−0.17 (−0.48, 0.14)	43.1%	2	0.08 (−0.34, 0.50)	0	2	0.23 (−0.11, 0.56)	0	2	0.54 (0.21, 0.88)^*^	0
Holladay 2 vs. SRK/T	4	0.21 (−0.02, 0.44)	0	2	−0.42 (−1.14, 0.31)	63.5%	2	0.32 (−0.01, 0.66)	0	2	0.60 (0.26, 0.93)^*^	0
Hoffer Q vs. SRK/T	5	0.21 (−0.19, 0.60)	79.5%	3	−0.22 (−0.68, 0.25)	66.2%	5	0.28 (0.06, 0.51)^*^	48.5%	4	0.09 (−0.20, 0.37)	76.1%
Hoffer Q vs. SRK II	3	−0.24 (−0.58, 0.11)	63.7%	NA			4	0.28 (−0.26, 0.82)	90.1%	3	−0.03 (−0.60, 0.55)	94.2%
SRK/T vs. SRK II	3	**−0.37 (−0.63**, −0.12)^*^	37.3%	NA			4	0.07 (−0.27, 0.42)	75.6%	3	−0.09 (−0.34, 0.17)	69.7%

*AL, axial length; MD, mean difference; 95% CI, 95% confidence interval; NA, not available*.

* *Indicates statistical significance*.

### Publication Bias

The publication bias was tested using Egger's linear regression test and the Begg's rank correlation test (Supplementary Table 3). The results did not show significant bias in any of the comparisons, which was consistent with the funnel plots (Supplementary Figures 3, 4).

## Discussion

In summary, the current meta-analysis included 12 studies involving 1,647 eyes with pediatric cataract, providing the most up-to-date and comprehensive evidence of the predictability levels of the different IOL power calculation formulas. Briefly, SRK/T formula exhibited a significantly smaller APE than Hoffer Q among the patients younger than 24 months. Among the patients aged 24–60 months, both SRK/T and Hoffer Q formulas were superior to Holladay 2, and SRK II was outperformed by Holladay 1. For patients with AL <22 mm, SRK/T and Holladay 1 showed a smaller APE than SRK II.

Despite the rapid development of biometrical measurement techniques, the accurate measurement of pediatric parameters remains challenging due to the poor cooperation by the children and their ever-changing eye conditions. Previous studies have indicated that the ALs of the children's eyes grew rapidly within the first 2 years and became relatively stable at the adult's level until the age of 5 (40–42). Other structures including ACD and corneal curvature, which are important parameters in calculating IOL power, also underwent significant changes in the first 42 months (42). Consequently, as previous studies have suggested, age, AL, keratometry, and ACD are the major factors affecting refractive error (21, 31, 32, 34). Indeed, the results of our meta-analysis based on all the included samples were characterized by substantial heterogeneity, which may be attributed to the different eye conditions of the patients included in the meta-analysis.

The postoperative refractive error was largely influenced by the AL measurement, postoperative ACD, and corneal power errors (43, 44). In our subgroup analysis, the AL subgroups were divided into <22 mm and 22–24.5 mm. On the basis of the previous evidence, the use of Holladay 2 and Hoffer Q for adult patients with short eyes has been recommended (11, 45). Our results indicated that among the patients with an AL <22 mm, the fourth-generation of Holladay 2 formula outperformed Hoffer Q. Similarly, in short eyes of adults, Hoffer Q has been reported to have better predictivity than Holladay 2 (11). A previous meta-analysis that focused on short eyes of adults found that Holladay 2, which, unlike other formulas, incorporates patients' additional biometrical data, produced the smallest mean APE (12). Furthermore, in accordance with the previous evidence, SRK II was found to be inferior to Holladay 1 and SRK/T in our meta-analysis (6, 8, 19). The older second-generation formula SRK II was based on a regression model and has been reported to provide the least accuracy (8, 10). For pediatric patients with a longer AL (22–24.5 mm), the third-generation SRK/T formula had a significantly smaller PE than Hoffer Q, with no heterogeneity observed, suggesting the robustness of the results. The results from adults also suggested that SRK/T outperformed Hoffer Q in eyes with a moderate AL (17).

The multiple linear regression analyses from previous studies have demonstrated that age is significantly associated with postoperative refractive error (21, 32, 35, 39). The current meta-analysis included pediatric patients aged 1.1–216 months, which covers the whole period of the eye development pf a child. As a child grows, the refractive status changes significantly due to the elongation of the AL, which mainly occurs in the first 2 years of life (46, 47). Therefore, the age subgroups were divided into younger than 24 months and 24–60 months. In the patients younger than 24 months, SRK/T was more accurate than Hoffer Q. The SRK/T formula is a non-linear theoretical formula empirically optimized for ACD, AL, retinal thickness, and corneal curvature (9). However, as a child ages, the expected significant myopic shift should be taken into account, and it is common to aim for hypermetropia instead of emmetropia, especially for children younger than 2 years old (47, 48). Therefore, the results of this study should be interpreted with caution, and the myopic shift after IOL implantation should be further considered. In the 24–60 months age subgroup, SRK/T and Hoffer Q showed a smaller APE than Holladay 2, and Holladay 1 showed a smaller APE than SRK II. Although no heterogeneity was observed, only two studies were included in each subgroup analysis, which precluded the reaching of a definitive conclusion. Taken together, a conclusion cannot be easily drawn, and age-specific IOL power calculation formulas for pediatric cataract patients should be further considered by the future studies.

Despite the potential errors of the IOL calculation formulas, eye parameters measurement deviation may also be a source of refractive error. For instance, lack of cooperation regarding precise fixation and centration, limited equipment designed for children's eyes, and the errors induced by the small size of pediatric eyes all contribute to the measurement inaccuracies (36, 38). Even under anesthesia, the measurement of keratometry was reported to be inaccurate due to lack of fixation (46). Therefore, more advanced measurement equipment designed specifically for pediatric patients are needed to achieve better accuracy.

Our meta-analysis evaluated and compared the predictability levels of the commonly applied IOL power calculation formulas in pediatric cataract patients. Nevertheless, the study was subject to several limitations. First, the pooled results were mainly based on retrospective cohort studies, which are subject to inevitable selection bias and confounding. We therefore assessed the risk of bias with QUADAS-2 and did not detect a significant risk of bias in the included studies. Second, there were moderate to substantial heterogeneity observed in some analyses. However, the sensitivity analyses indicated the study by Kekunnaya et al. (21) is the source of heterogeneity, and confirmed the stability of the results. Kekunnaya et al. (21) included data only from children younger than 24 months, which may partially explain the heterogeneity. Third, due to the significant variability of the study sample, we failed to reach a definitive conclusion for the whole study sample. However, the subgroup analyses based on age and AL have provided significant and meaningful results.

In summary, the present meta-analysis demonstrated high variability of refractive status among pediatric patients. Among the various IOL power calculation formulas currently available, SRK/T presented a relatively smaller postoperative refractive error under certain conditions. In real clinical practice, the clinical judgment should be based on the characteristics of the patient, the features of the formulas used, and the surgeon's experience. For pediatric cataract patients, more evidence-based and age-specific publications are needed to provide clinical guidelines for formula selection and accurate IOL power calculation.

## Data Availability Statement

The original contributions presented in the study are included in the article/Supplementary Material, further inquiries can be directed to the corresponding author.

## Author Contributions

YZhu conceived and designed the study. YZhong and YY performed literature search and data collection. YZhong, YY, and JL performed statistical analysis. YZhong, BL, and SL drafted the manuscript. YZhu revised the manuscript. All authors approved the final version to be published and agreed to be accountable for all aspects of the work.

## Funding

This work was supported by the Program of National Natural Science Foundation of China (Grant No. 81970779 to YZhu), the Program of National Natural Science Foundation of China (82070938 to YY), and the Program of National Natural Science Foundation of Zhejiang Province (LY20H120011 to YY).

## Conflict of Interest

The authors declare that the research was conducted in the absence of any commercial or financial relationships that could be construed as a potential conflict of interest.

## Publisher's Note

All claims expressed in this article are solely those of the authors and do not necessarily represent those of their affiliated organizations, or those of the publisher, the editors and the reviewers. Any product that may be evaluated in this article, or claim that may be made by its manufacturer, is not guaranteed or endorsed by the publisher.
