# Supplementary material for: Accuracy of Intraocular Lens Power Calculation Formulas in Pediatric Cataract Patients: A Systematic Review and Meta-Analysis
Source: Front Med (Lausanne). 2021 Nov 26;8:710492. doi: 10.3389/fmed.2021.710492 (PMC8661900; doi:10.3389/fmed.2021.710492)
Supplement: Supplementary file 1 [file Data_Sheet_1.docx]

Supplementary Material

# Supplementary Information

**Supplementary Table 1.** QUADAS-2 assessment of risk of bias (n=12).

**Supplementary Table 2.** Sensitivity analyses by omitting a single study for prediction error (PE) and absolute prediction error (APE).

**Supplementary Table 3.** Publication bias measured by Begg’s and Egger’s test.

**Supplementary Figure 1.** The overall absolute prediction error (APE) of each intraocular lens (IOL) power calculation formula.

**Supplementary Figure 2.** Forest plots of absolute prediction error (APE) between different formula after excluding Li et al. (**A, C**)*.* and Kekunnaya et al.(**B**).

**Supplementary Figure 3.** Funnel plots of included studies for prediction error (PE).

**Supplementary Figure 4.** Funnel plots of included studies for absolute prediction error (APE).

# Supplementary Figures and Tables

## Supplementary Tables

**Supplementary Table 1.** QUADAS-2 assessment of risk of bias (n=12).

| **Author, year** | **Risk of bias** | | | |  | **Applicability concerns** | | |
| --- | --- | --- | --- | --- | --- | --- | --- | --- |
|  | **Patient selection** | **Index test** | **Reference standard** | **Flow and timing** |  | **Patient selection** | **Index test** | **Reference standard** |
| Eppley, 2021 | Low | Low | Unclear | High |  | Low | Low | Unclear |
| Chang, 2020 | Low | Low | Unclear | Low |  | Low | Low | Unclear |
| Kou, 2020 | Low | Low | Unclear | Low |  | Low | Low | Unclear |
| Li, 2020 | Low | Low | Unclear | High |  | Low | Low | Unclear |
| Lee, 2019 | High | Low | Unclear | Low |  | Low | Low | Unclear |
| Vasavada, 2016 | Low | Low | Unclear | Low |  | Low | Low | Unclear |
| Joshi, 2014 | High | Low | Unclear | High |  | Low | Low | Unclear |
| Vanderveen, 2013 | High | Low | Unclear | Low |  | Low | Low | Unclear |
| Kekunnaya, 2012 | Low | Low | Unclear | Low |  | Low | Low | Unclear |
| Trivedi, 2011 | Low | Low | Unclear | High |  | Low | Low | Unclear |
| Nihalani, 2010 | Low | Low | Unclear | Low |  | Low | Low | Unclear |
| Mezer, 2003 | High | Low | Unclear | High |  | Low | Low | Unclear |

**Abbreviations:** QUADAS-2, Quality Assessment of Diagnostic Accuracy Studies-2.

**Supplementary Table 2.** Sensitivity analyses by omitting a single study for PE and APE.

| **Formula comparison** | **PE** | |  | **APE** | |  |
| --- | --- | --- | --- | --- | --- | --- |
|  | ***I*^2^ (%)** | **MD (95% CI)** |  | ***I*^2^ (%)** | **MD (95% CI)** |  |
| **Holladay 1 vs. Holladay 2** |  |  |  |  |  |  |
| Chang, 2020 | 20.7% | -0.06 (-0.32, 0.19) |  | 0 | 0.01 (-0.18, 0.20) |  |
| Vasavada, 2016 | 72.0% | 0.02 (-0.48, 0.52) |  | 0 | -0.14 (-0.37, 0.08) |  |
| Vanderveen, 2013 | NA | |  | 20.3% | -0.05 (-0.26, 0.16) |  |
| Trivedi, 2011 | 16.1% | 0.12 (-0.11, 0.34) |  | 14.6% | -0.09 (-0.29, 0.11) |  |
| **Holladay 1 vs. Hoffer Q** |  |  |  |  |  |  |
| Chang, 2020 | 64.9% | -0.03 (-0.21, 0.15) |  | 41.9% | -0.12 (-0.25, 0.01) |  |
| Li, 2020 | 61.8% | -0.10 (-0.31, 0.11) |  | 21.7% | **-0.15 (-0.28, -0.02)*** |  |
| Vasavada, 2016 | 66.1% | -0.04 (-0.24, 0.15) |  | 42.4% | -0.11 (-0.25, 0.03) |  |
| Joshi, 2014 | 65.8% | -0.03 (-0.21, 0.15) |  | 40.4% | -0.10 (-0.23, 0.03) |  |
| Vanderveen, 2013 | NA | |  | 10.8% | -0.07 (-0.17, 0.03) |  |
| Kekunnaya, 2012 | 44.0% | -0.11 (-0.26, 0.05) |  | 0 | -0.04 (-0.13, 0.05) |  |
| Trivedi, 2011 | 64.5% | -0.03 (-0.20, 0.15) |  | 42.4% | -0.12 (-0.25, 0.01) |  |
| Nihilani, 2010 | 64.6% | -0.04 (-0.23, 0.16) |  | 40.2% | -0.13 (-0.27, 0.01) |  |
| Mezer, 2003 | NA | |  | 42.0% | -0.12 (-0.25, 0.01) |  |
| **Holladay 1 vs. SRK/T** |  |  |  |  |  |  |
| Chang, 2020 | 39.8% | 0.01 (-0.13, 0.14) |  | 3.0% | -0.01 (-0.11, 0.08) |  |
| Li, 2020 | 18.1% | 0.05 (-0.09, 0.19) |  | 0 | 0.04 (-0.07, 0.15) |  |
| Vasavada, 2016 | 37.4% | 0.001 (-0.140, 0.143) |  | 0.6% | -0.02 (-0.11, 0.08) |  |
| Joshi, 2014 | 37.0% | 0.0003 (-0.1279, 0.1285) |  | 1.0% | -0.01 (-0.10, 0.08) |  |
| Vanderveen, 2013 | NA | |  | 0 | -0.02 (-0.11, 0.07) |  |
| Kekunnaya, 2012 | 16.3% | 0.04 (-0.08, 0.15) |  | 0 | -0.03 (-0.13, 0.06) |  |
| Trivedi, 2011 | 24.1% | -0.02 (-0.13, 0.10) |  | 0 | 0.002 (-0.088, 0.092) |  |
| Nihilani, 2010 | 24.8% | -0.02 (-0.15, 0.11) |  | 0 | 0.01 (-0.08, 0.10) |  |
| Mezer, 2003 | NA | |  | 7.6% | 0.0002 (-0.0971, 0.0976) |  |
| **Holladay 1 vs. SRK II** |  |  |  |  |  |  |
| Chang, 2020 | 93.9% | -0.03 (-0.53, 0.48) |  | 91.1% | -0.04 (-0.42, 0.34) |  |
| Li, 2020 | 94.1% | 0.11 (-0.53, 0.75) |  | 89.6% | -0.02 (-0.44, 0.41) |  |
| Joshi, 2014 | 93.2% | -0.12 (-0.56, 0.32) |  | 89.9% | -0.15 (-0.49, 0.20) |  |
| Vanderveen, 2013 | NA | |  | 90.8% | -0.01 (-0.37, 0.36) |  |
| Kekunnaya, 2012 | 89.2% | 0.22 (-0.20, 0.63) |  | 58.1% | **-0.23 (-0.43, -0.04)*** |  |
| Nihilani, 2010 | 91.7% | -0.06 (-0.54, 0.42) |  | 90.0% | 0.004 (-0.381, 0.388) |  |
| Mezer, 2003 | NA | |  | 91.1% | -0.05 (-0.43, 0.33) |  |
| **Holladay 2 vs. Hoffer Q** |  |  |  |  |  |  |
| Eppley, 2021 | 29.6% | **-0.228 (-0.453, -0.003)*** |  | NA | |  |
| Chang, 2020 | 0 | -0.09 (-0.28, 0.09) |  | 0 | -0.18 (-0.38, 0.01) |  |
| Vasavada, 2016 | 49.7% | -0.18 (-0.48, 0.11) |  | 64.8% | -0.04 (-0.43, 0.34) |  |
| Vanderveen, 2013 | NA | |  | 47.9% | 0.01 (-0.26, 0.27) |  |
| Trivedi, 2011 | 44.4% | -0.21 (-0.45, 0.04) |  | 68.7% | -0.08 (-0.43, 0.26) |  |
| **Holladay 2 vs. SRK/T** |  |  |  |  |  |  |
| Eppley, 2021 | 71.0% | 0.10 (-0.26, 0.45) |  | NA | |  |
| Chang, 2020 | 42.7% | 0.19 (-0.06, 0.45) |  | 52.3% | 0.05 (-0.25, 0.35) |  |
| Vasavada, 2016 | 71.3% | 0.14 (-0.26, 0.53) |  | 65.6% | 0.17 (-0.22, 0.55) |  |
| Vanderveen, 2013 | NA | |  | 59.4% | 0.06 (-0.24, 0.36) |  |
| Trivedi, 2011 | 11.7% | 0.002 (-0.187, 0.191) |  | 34.0% | 0.22 (-0.02, 0.45) |  |
| **Hoffer Q vs. SRK/T** |  |  |  |  |  |  |
| Eppley, 2021 | 89.7% | 0.17 (-0.09, 0.42) |  | NA | |  |
| Chang, 2020 | 89.5% | 0.15 (-0.11, 0.41) |  | 81.9% | 0.18 (-0.02, 0.38) |  |
| Li, 2020 | 83.5% | 0.21 (-0.02, 0.45) |  | 69.9% | **0.21 (0.04, 0.39)*** |  |
| Lee, 2019 | 84.2% | 0.13 (-0.12, 0.38) |  | 76.2% | 0.15 (-0.05, 0.35) |  |
| Vasavada, 2016 | 89.6% | 0.16 (-0.11, 0.43) |  | 81.9% | 0.17 (-0.03, 0.37) |  |
| Joshi, 2014 | 89.4% | 0.13 (-0.11, 0.38) |  | 81.5% | 0.15 (-0.03, 0.34) |  |
| Vanderveen, 2013 | NA | |  | 78.5% | 0.12 (-0.05, 0.31) |  |
| Kekunnaya, 2012 | 83.1% | **0.25 (0.03, 0.46)*** |  | 77.6% | 0.13 (-0.05, 0.31) |  |
| Trivedi, 2011 | 89.1% | 0.12 (-0.12, 0.37) |  | 80.9% | **0.20 (0.01, 0.39)*** |  |
| Nihilani, 2010 | 89.4% | 0.15 (-0.12, 0.41) |  | 79.4% | **0.21 (0.02, 0.40)*** |  |
| Mezer, 2003 | NA | |  | 81.6% | 0.190 (-0.004, 0.384) |  |
| **Hoffer Q vs. SRK II** |  |  |  |  |  |  |
| Chang, 2020 | 97.6% | 0.12 (-0.48, 0.71) |  | 95.0% | 0.17 (-0.22, 0.57) |  |
| Li, 2020 | 96.6% | 0.27 (-0.35, 0.89) |  | 92.4% | 0.19 (-0.19, 0.57) |  |
| Lee, 2019 | 96.0% | 0.09 (-0.50, 0.67) |  | 93.3% | 0.08 (-0.33, 0.49) |  |
| Joshi, 2014 | 97.5% | 0.02 (-0.54, 0.57) |  | 94.9% | 0.05 (-0.33, 0.42) |  |
| Vanderveen, 2013 | NA | |  | 95.2% | 0.13 (-0.27, 0.52) |  |
| Kekunnaya, 2012 | 95.8% | 0.39 (-0.07, 0.86) |  | 92.5% | -0.002 (-0.336, 0.332) |  |
| Nihilani, 2010 | 97.6% | 0.11 (-0.51, 0.73) |  | 94.2% | 0.21 (-0.17, 0.59) |  |
| Mezer, 2003 | NA | |  | 95.1% | 0.16 (-0.23, 0.56) |  |
| **SRK/T vs. SRK II** |  |  |  |  |  |  |
| Chang, 2020 | 89.1% | 0.01 (-0.27, 0.29) |  | 85.5% | -0.06 (-0.29, 0.18) |  |
| Li, 2020 | 87.2% | 0.08 (-0.24, 0.40) |  | 83.8% | -0.07 (-0.33, 0.20) |  |
| Lee, 2019 | 85.4% | 0.01 (-0.30, 0.31) |  | 83.8% | -0.12 (-0.38, 0.15) |  |
| Joshi, 2014 | 88.2% | -0.03 (-0.28, 0.23) |  | 85.3% | -0.13 (-0.35, 0.10) |  |
| Vanderveen, 2013 | NA | |  | 83.1% | -0.02 (-0.24, 0.19) |  |
| Kekunnaya, 2012 | 74.0% | 0.14 (-0.06, 0.33) |  | 74.5% | -0.17 (-0.36, 0.02) |  |
| Nihilani, 2010 | 88.6% | 0.001 (-0.288, 0.290) |  | 84.2% | -0.05 (-0.29, 0.19) |  |
| Mezer, 2003 | NA | |  | 86.1% | -0.08 (-0.32, 0.16) |  |

## Abbreviation: PE, prediction error; APE, absolute prediction error; MD, mean difference; 95% CI, 95 confidence intervals; NA, not available.

***** Indicates statistical significance.

**Supplementary Table 3.** Publication bias measured by Begg’s and Egger’s test.

| **Formula comparison** | **PE** | |  | **APE** | |
| --- | --- | --- | --- | --- | --- |
|  | **Begg’s test** | **Egger’s test** |  | **Begg’s test** | **Egger’s test** |
| Holladay 1 vs. Holladay 2 | 1.00 | 0.77 |  | 0.73 | 0.64 |
| Holladay 1 vs. Hoffer Q | 0.37 | 0.13 |  | 0.12 | 0.18 |
| Holladay 1 vs. SRK/T | 0.37 | 0.10 |  | 0.47 | 0.20 |
| Holladay 1 vs. SRK II | 0.81 | 0.42 |  | 0.23 | 0.53 |
| Holladay 2 vs. Hoffer Q | 0.73 | 0.90 |  | 0.73 | 0.91 |
| Holladay 2 vs. SRK/T | 0.31 | 0.48 |  | 1.00 | 0.87 |
| Hoffer Q vs. SRK/T | 0.75 | 0.75 |  | 1.00 | 0.76 |
| Hoffer Q vs. SRK II | 1.00 | 0.99 |  | 0.39 | 0.93 |
| SRK/T vs. SRK II | 1.00 | 0.91 |  | 1.00 | 0.74 |

**Abbreviations:** PE, prediction error; APE, absolute prediction error.

## Supplementary Figures


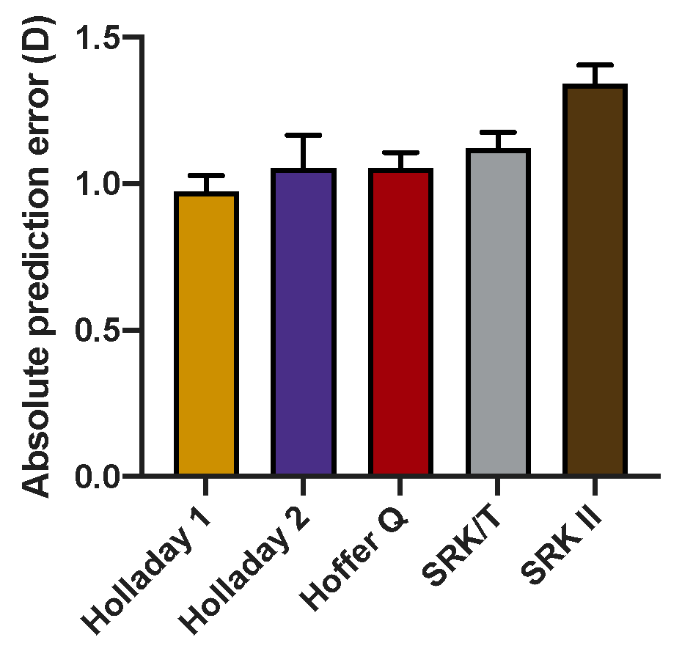


**Supplementary Figure 1.** The overall absolute prediction error (APE) of each intraocular lens (IOL) power calculation formula.


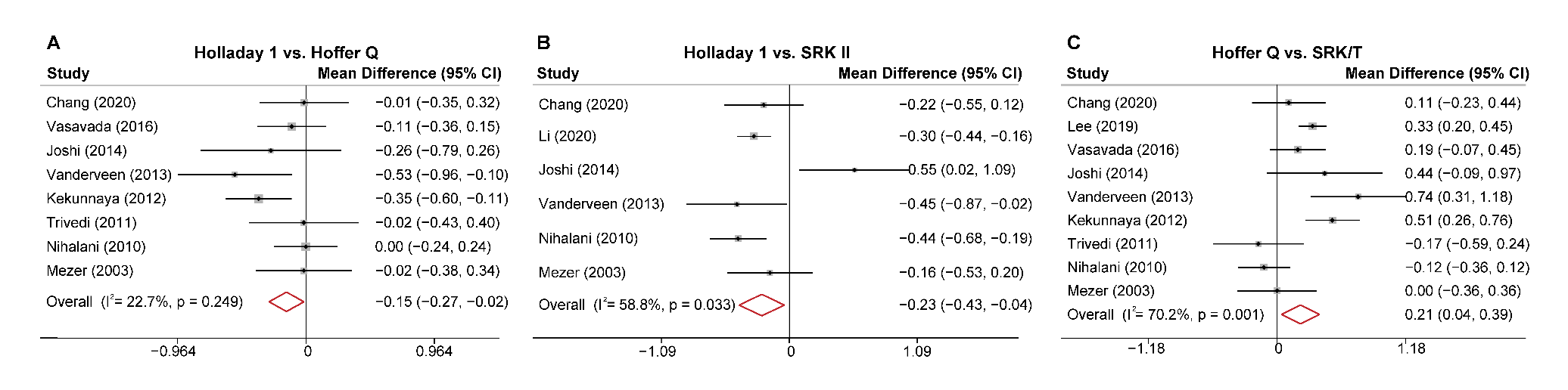


**Supplementary Figure 2.** Forest plots of absolute prediction error (APE) between different formula after excluding Li *et al* (**A, C**)*.* and Kekunnaya *et al.*(**B**).

**
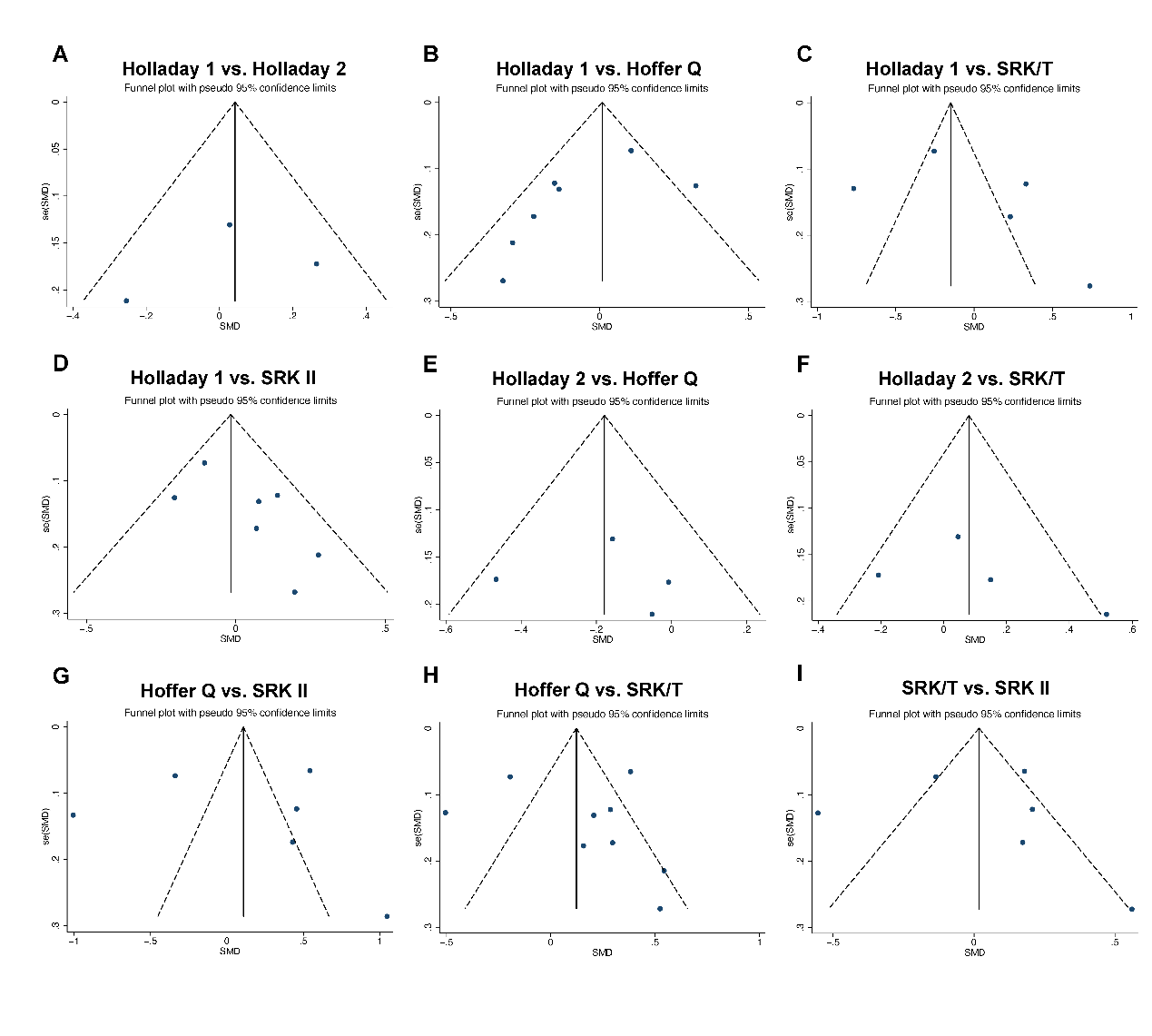
**

**Supplementary Figure 3.** Funnel plots of included studies for prediction error (PE).

**
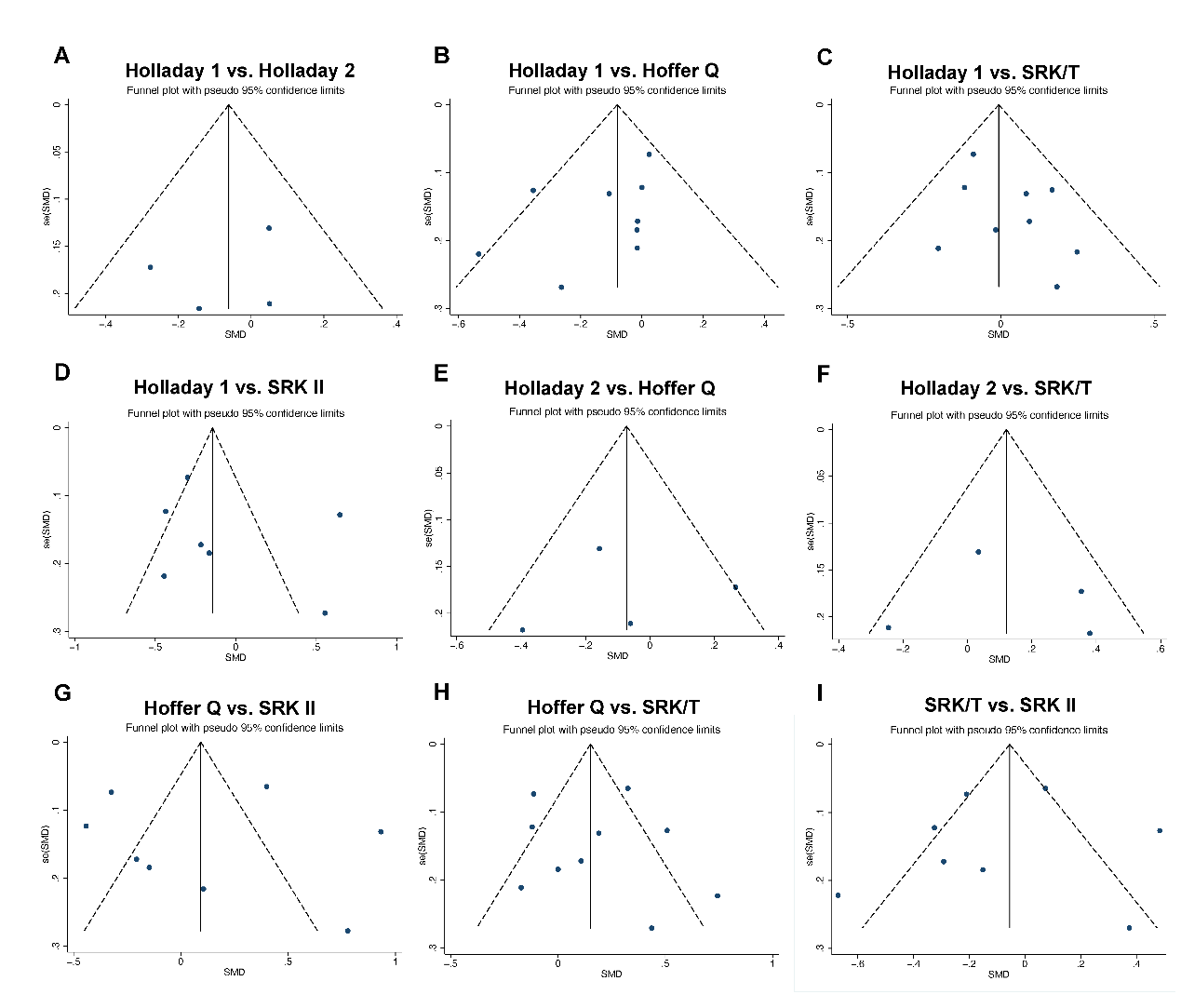
**

**Supplementary Figure 4.** Funnel plots of included studies for absolute prediction error (APE).
